# A pipeline for RNA-seq based eQTL analysis with automated quality control procedures

**DOI:** 10.1186/s12859-021-04307-0

**Published:** 2021-08-25

**Authors:** Tao Wang, Yongzhuang Liu, Junpeng Ruan, Xianjun Dong, Yadong Wang, Jiajie Peng

**Affiliations:** 1 School of Computer Science, Northwestern Polytechnical University, 1 Dongxiang Road, Chang’an District, Xi’an, China; 2 School of Computer Science and Technology, Harbin Institute of Technology, West Dazhi St., Harbin, China; 3 Brigham and Women’s Hospital, Harvard Medical School, 75 Francis St., Boston, USA

## Abstract

**Background:**

Advances in the expression quantitative trait loci (eQTL) studies have provided valuable insights into the mechanism of diseases and traits-associated genetic variants. However, it remains challenging to evaluate and control the quality of multi-source heterogeneous eQTL raw data for researchers with limited computational background. There is an urgent need to develop a powerful and user-friendly tool to automatically process the raw datasets in various formats and perform the eQTL mapping afterward.

**Results:**

In this work, we present a pipeline for eQTL analysis, termed eQTLQC, featured with automated data preprocessing for both genotype data and gene expression data. Our pipeline provides a set of quality control and normalization approaches, and utilizes automated techniques to reduce manual intervention. We demonstrate the utility and robustness of this pipeline by performing eQTL case studies using multiple independent real-world datasets with RNA-seq data and whole genome sequencing (WGS) based genotype data.

**Conclusions:**

eQTLQC provides a reliable computational workflow for eQTL analysis. It provides standard quality control and normalization as well as eQTL mapping procedures for eQTL raw data in multiple formats. The source code, demo data, and instructions are freely available at https://github.com/stormlovetao/eQTLQC.

## Introduction

With the development of genome-wide assay of genetic variants, vast of complex traits associated variants have been detected by the genome-wide association studies (GWAS) [1]. However, the function of GWAS signals largely remains elusive, because most GWAS-derived variants locate in the non-coding regions, i.e. intergenic or intronic regions, which means they do not alter the protein sequence directly [2]. Understanding the function of variants associated with diseases and other traits has been one of the focuses in the field of the post-GWAS era, which could benefit the discovery of novel mechanisms and drug targets [3–5]. Emerging evidence has shown that variants could exert their effects by regulating expression levels of local or distant genes, which are termed as expression quantitative trait loci (eQTL). The eQTL analysis aims to associate genetic variants with the variation of gene expression levels. The eQTL summaries have been widely applied in interpreting GWAS and in mendelian randomization studies [6, 7].

As the decrease of cost in next-generation sequencing (NGS), RNA-seq technology has been widely used to measure the presence and quantity of transcriptome in eQTL studies [8]. RNA-seq advances microarray in multiple aspects. First, RNA-seq has higher sensitivity and accuracy towards low-abundance transcripts. Second, RNA-seq could cover all transcripts in theory, both coding and non-coding RNAs, while microarray can only measure annotated transcripts with certain abundance [9]. For example, in our recent work, we performed total RNA sequencing (sequencing RNA with or without Ploy-A tail) of dopamine neurons in substantia nigra, and this enabled us to detect eQTLs regulating enhancer RNA [2]. Furthermore, RNA-seq can be used to detect the splicing events of pre-mRNA, which enables the research of splicing QTL(sQTL). To perform eQTL analysis, RNA-seq data is required to be processed into an expression matrix. From RNA-seq reads to the expression matrix, multiple quality control (QC) and normalization steps are needed to remove biases from samples, techniques, or artificial factors. Besides the RNA-seq data, genotypes of the same samples are also required to perform eQTL analysis. And rigorous quality control procedures of raw genotyping data are also needed to enable high-quality eQTL analysis.

The rapid development of tools and databases helps researchers to understand complex diseases [10–15]. In recent years, multiple advanced eQTL analysis tools have been developed, such as MatrixEQTL [16], FastQTL [17] and QTLTools [18], which have different outstanding features. MatrixEQTL achieves fast computing efficiency by taking advantage of large matrix operations [16]. FastQTL efficiently controls the multiple permutation testing problem [17]. QTLTools integrates multiple tools to perform molecular QTL discovery and downstream functional annotation analysis [18]. However, few tools can automatically pre-process the genotype data and gene expression data, which are necessary for eQTL analysis. Although published protocols exist for the processing of RNA-seq data and genotype data respectively [9, 19, 20], there is currently no computational workflow that can be compatible with multi-source heterogeneous eQTL raw data. And it also remains a big challenge for users who have limited computational backgrounds to deal with vast preprocessing details. Thus, there is an urgent need to develop an automatic eQTL analysis tool involving standard and rigorous quality control procedures for eQTL studies. To achieve this purpose, several challenges need to be addressed. First, various data formats and data types exist in eQTL raw data. For example, depending on the stage of RNA-seq analysis, gene expression data may be in FASTQ, BAM, or read count formats. Second, biases commonly exist in both RNA-seq and genotype data, and comprehensive quality control and normalization procedures are required. Besides, in the current preprocessing protocol, a large amount of effort is required to manually run each step, which may also induce biases. Therefore, tools that can automatically handle computing details are needed to reduce manual intervention.

In this study, we present an automated pipeline for eQTL analysis, termed eQTLQC, featured with a set of rigorous preprocessing approaches for both genotype and gene expression data. The main designs of eQTLQC have been used in data preprocessing and eQTLs identification in our recent work [2, 21]. To aid the automatic process, eQTLQC provides users with a JSON configuration file to setup parameters and the processing logic flexibly. Our eQTL analysis framework supports various input formats for RNA-seq and genotype data. Machine learning-based and empirical techniques are used in the workflow to reduce manual intervention. In the following context, we demonstrate the utility and feasibility of eQTLQC by performing an eQTL case study using real-world datasets generated by ROSMAP studies [22, 23].

## Results

**Fig. 1:**
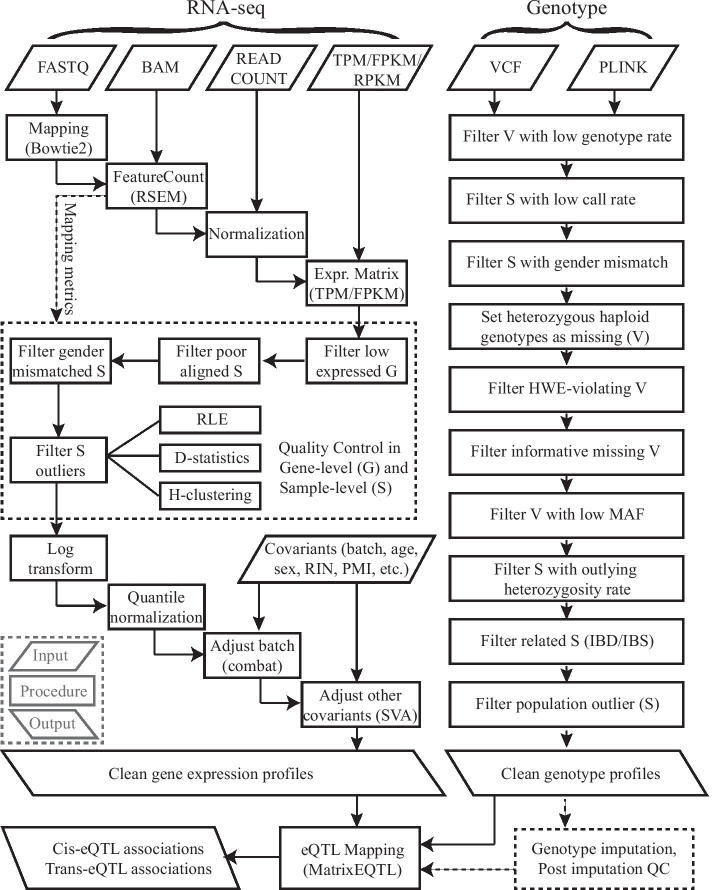
Workflow. Framework of eQTLQC. Abbreviations: sample (S); gene (G); variants (V); Hardy–Weinberg equilibrium (HWE); identical by state (IBS); identical by descent (IBD); minor allele frequency (MAF); Post-mortem interval (PMI); RNA integrity number (RIN); relative log expression (RLE); hierarchical clustering (H-clustering)

As shown in Fig. 1, we present a computational framework for data preprocessing and eQTL analysis. Rigorous quality control and normalization procedures are applied to gene expression data and genotype data, followed by standard eQTL mapping. In the following sections, we utilize this framework to process data from ROSMAP study [22, 23], and also three other datasets for the purpose of robustness analysis, including MayoRNAseq (Mayo) [24], MSBB [25] and CommonMind [26].

### Preprocessing of gene expression data

#### Harmonization of multi-source heterogeneous eQTL raw data

RNA-seq based gene expression data is usually generated in the following four RNA-seq processing stages and in different data formats: the raw reads stage (FASTA/FASTQ formats), the read-aligned stage (SAM/BAM formats), feature-counted stage (read count format), or the standardized stage (RPKM/FPKM/TPM formats). The eQTL raw data could be at any of the four stages. And these data formats are heterogeneous and require different processing steps before eQTL mapping analysis.

The eQTLQC integrates several standard tools and in-house scripts to transform the data formats in the first three stages into the format of standardized stage (i.e. TPM). For expression data in FASTA/FASTQ formats, eQTLQC provides Bowtie [27], Bowtie2 [28] and Star [29] for reads alignment, and generates BAM files. For expression data in SAM/BAM formats, eQTLQC utilizes the rsem-calculate-expression function in RSEM [30] to generate gene read counts. For expression data in read count format, we use in-house script to transform it into TPM formats. For data in the standardized stage, eQTLQC will perform rigorous quality control and normalization steps which will be described in the following context.

To illustrate the following steps, we will perform an eQTL case study starting from the gene read count table from ROSMAP study [22, 23], representing 60,554 genes and 370 neuropathologically healthy samples with clinical consensus diagnosis score $\le 3$ (no/mild cognitive impairment). Three main function modules are implemented in eQTLQC, including (1) basic quality control module, which includes transforming read counts into TPM values, removing genes with low expression levels and excluding gender-mismatched samples; (2) detecting and removing sample outliers with problematic gene expression profiles; (3) quantile normalization and adjusting covariates of gene expression profiles.

#### Basic quality control on gene read counts

We first normalize read counts by gene length and sequencing library depth within-sample using TPM transformation. The TPM value of a gene reflects the relative transcription abundance of this gene in a sample by measuring how many RNA molecules are derived from this gene in every million RNA molecules. The TPM transformation has been widely used in eQTL studies, such as GTEx study [8]. To be noted, we recommend users use the length of the union of exons as the gene length when performing TPM transformation. After TPM transformation, we identify and exclude genes with low expression levels. Users could exclude genes with TPM $< a$ in $\ge b\%$ samples or genes with less than *c* reads in $\ge d\%$ samples. Besides, users could only keep samples with $>e$ million mapped reads and with $>f\%$ mappability. These parameters can be personalized using a configuration file in JSON format, and same as other parameters in the following context. By setting $a = 0.1, b = d = 80, c = 6, e = 10, f = 70$, there are 367 samples and 26,662 genes left after removing low-expressed genes or poor-aligned samples in ROSMAP.

**Fig. 2:**
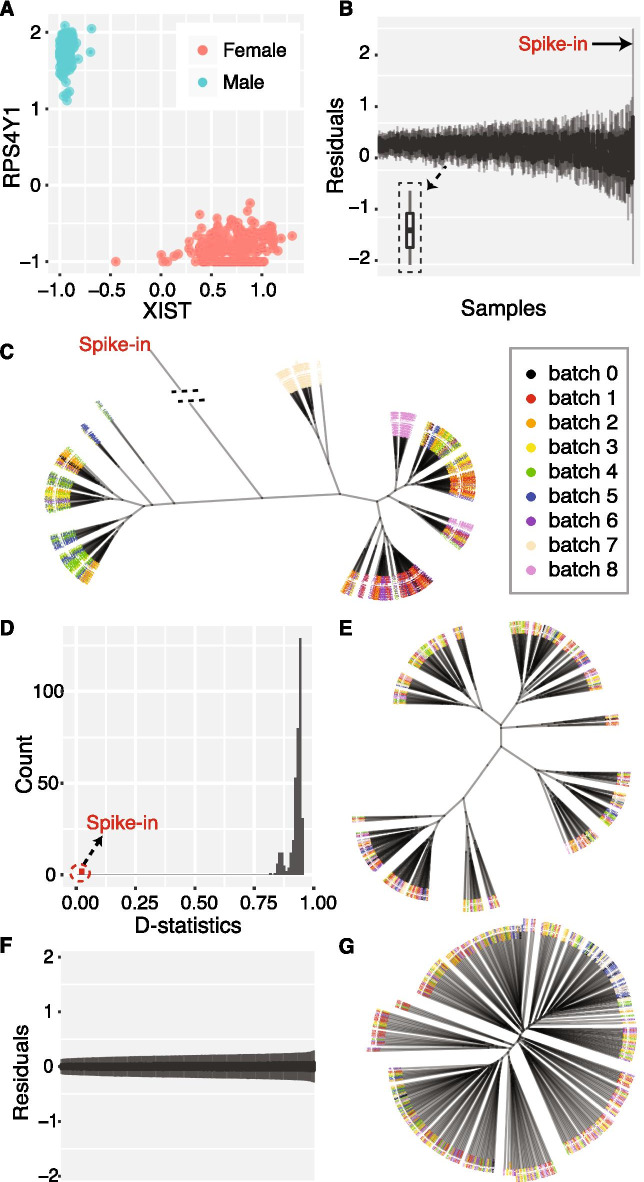
Expr_QC. Identification of outlying samples and normalization of RNA-seq based gene expression data. **a** Gene expression levels of two gender-specific genes: RPS4Y1 and XIST. **b** The RLE plot before data preprocessing. Aligned box-plots, in increasing order of IQR, represent residuals of gene expression for samples. **c** Hierarchical clustering of samples before data preprocessing. Color represents the batch of library preparation. **d** Distribution of D-statistics before data preprocessing. **e** Hierarchical clustering dendrogram after adjusting batch effects. **f** The RLE plot after data preprocessing. **g** Hierarchical clustering dendrogram after data preprocessing

Next, we identify samples with mismatched gender, which might be due to sample swap or mix-up. To achieve this purpose, we first predict the gender of the sample donator by measuring the expression profiles of two gender-specific genes, RPS4Y1 and XIST, which are specifically expressed in males and females respectively. Then, we compare the predicted gender and user-given gender to identify mismatched samples. Usually, the gender-mismatched samples are identified manually based on the scatter plot. To avoid artificial biases, we automatically identify the abnormal samples using the support vector machine (SVM) classifier. Under the hypothesis that only a few samples have incorrect gender labels, we first train the SVM model using the expression profiles of RPS4Y1 and XIST as features and the given gender labels. Then, we predict the sample labels based on the trained model. Samples will be excluded if the predicted labels are different from the user-given labels. In the ROSMAP dataset, we did not detect gender-mismatched samples, as shown in Fig. 2a.

#### Exclude sample outliers with problematic gene expression profile

Samples with problematic gene expression profiles might be caused by sample contamination or failure of library preparation or RNA sequencing etc. In our pipeline, we use three approaches to measure the distance between samples and identify sample outliers, including Relative Log Expression (RLE) analysis, pair-wise correlation-based hierarchical clustering, and D-statistics analysis [9]. A spike-in sample with problematic gene expression profiles is manually added, generated by randomly permuting mean gene expression values of existing samples.

The RLE analysis is under the hypothesis that in a sample only a few genes have extreme expression levels, while most genes have similar expression levels across samples. Given a gene expression matrix *G* with genes in row and samples in column, the RLE analysis first subtracts the median expression levels of each gene from the original expression value of all samples in each row. For each sample, the residual expression values of all genes should be centered at zero under the above hypothesis. Then, the RLE analysis makes boxplots for all samples based on residual gene expression values and sorts those boxplots or samples by the interquartile range (IQR) in increasing order from left to right, as shown in Fig. 2b. And samples at the rightmost part are likely to be outliers if they have an obvious larger IQR than other samples. As shown in Fig. 2b, the spike-in sample has the largest IQR in the RLE analysis. In eQTLQC, users can set the rightmost $x\%$ samples as candidate outliers.

Hierarchical clustering is also a widely used approach to exclude sample outliers. The similarity between each pair of samples is first measured by metrics such as Pearson’s correlation coefficient or Spearman’s correlation coefficient. The distance matrix, obtained by one minus similarity scores, is used to perform hierarchical clustering. Usually, samples with problematic expression profiles will be far from normal samples in the clustering dendrogram. As shown in Fig. 2c, we can see the spike-in sample is isolated in the dendrogram. Traditionally, the sample outliers will be manually picked out and excluded. To make this process automatic, we use a Mahalanobis distance-based approach to identify outliers, which has been applied in GTEx project [8]. The Mahalanobis distance measures the distance between a point *P* and a distribution *D* in multi-dimensional space. In our context, we measure the Mahalanobis distances between each sample and the distribution of all samples. The chi-squared *p* value is calculated for each sample in each cluster. Clusters with $\le y\%$ samples with Bonferroni-corrected *p* values $\le 0.05$ will be marked as outlier clusters, and all samples included will be marked as candidate outliers. In the ROSMAP dataset, the spike-in sample is clustered into a single cluster with itself only and achieves the most significant adjusted *p* value ($3.4*10^{-25}$), which should be excluded. And other clusters are not labeled as outliers based on the clustering analysis.

The third method to identify outliers is based on the distribution of D-statistics. The D-statistic of each sample is defined as the median Spearman’s correlation coefficients with other samples. Fig. 2d shows the distribution of D-statistics of all samples in the ROSMAP dataset, and samples locating at the leftmost tail of the distribution are likely to be outliers. We can see that the spike-in sample locates far from the peak of the distribution. In eQTLQC, users can set the leftmost $z\%$ samples as candidate outliers.

Combining the analyses of above, our pipeline considers the intersection of candidate outliers reported by the three methods as the final set of sample outliers. In the ROSMAP dataset, by setting $x=z=5, y = 40$, only the spike-in sample is labeled as outlier and excluded.

#### Normalization and covariates adjustment

To eliminate technical noises existing in RNA-seq experiment, we first perform within-sample inverse normal transformation by transforming TPM values to rank normalized values [2, 8]. In brief, the TPM values are first log10-transformed (adding a pseudocount of $10^{-4}$). Then, the measurements for each gene are transformed into normally distributed while preserving relative rankings (quantile normalization) and the mean and standard deviation of the original measurement.

We also perform cross-sample normalization to adjust known and latent covariates, which could bias the eQTL analysis. Common known covariates include technical artifacts such as batch effects, post-mortem interval (PMI) and RNA integrity number (RIN); and sample-relevant characteristics such as age, sex, and education length. Latent covariates such as environmental factors and population stratification are usually hard to access. In eQTLQC, we use SVA to adjust the known and hidden covariates. To be specified, we use the *combat* function in SVA [31] to adjust the batch effects, and use the *fsva* function to adjust other known covariates and latent covariates. In the ROSMAP dataset, we adjusted age, sex, batch, PMI, RIN, and 22 surrogate variables detected by SVA.

Figure 2c shows the clustering dendrogram of samples before performing normalization and covariates adjustment, where node color represents the batch information. We can see obvious batch effects where samples within the same batch tend to be in the same clusters. After the normalization and covariates adjustment, samples do not tend to be grouped according to batches as shown in Fig. 2e. Figure 2f and g show the RLE plot and clustering dendrogram respectively after the whole data preprocessing. We can see the improvement of data quality comparing with Fig. 2b and c, which are prior data preprocessing.

### Quality control of genotype data

Rigorous quality control of genotype data is also critical in eQTL analysis. eQTLQC accepts genotype data in VCF or PLINK formats, which are most widely used. Ten rigorous QC steps in both the SNP level and sample level are applied to improve data quality, as shown in Fig. 1. The QC procedures are based on published protocols and have been widely used in GWAS [32, 33] and eQTL studies [8], including our recent work [2]. Parameters were empirically set by default following the published protocols and can be adjusted by users in the configuration file. PLINK [34] will be used for performing the QC procedures, and VCF files will be transformed into PLINK format once provided. The genotype data of ROSMAP study are derived from WGS, consisting of 7,346,574 markers (SNPs and small Indels). Genotypes of 343 subjects whose RNA-seq data are also available will be used in the following QC procedures.

*Step 1: remove markers with excessive missing genotypes.* The genotype missing rate reflects the data quality. Variants with systematically missing genotype values have no help toward the downstream analysis and might cause false-positive signals [19]. In eQTLQC, variants with genotype missing rate greater than or equal to 5% will be excluded by default. In the ROSMAP genotype dataset, 147,989 variants are excluded in this step.

*Step 2: exclude subjects with excessive missing genotypes.* Similar to *Step 1*, the genotype missing rate in subject-level should also be checked. Subjects with a high genotype missing rate may be due to poor quality of DNA samples or library preparation. In the case that genotype data is generated and combined from different sequencing platforms or microarray chips, the missing genotypes are also very common in samples. In eQTLQC, subjects with genotype missing rate $<5\%$ will be left for further analysis. No subjects with excessive missing genotypes are excluded from the ROSMAP genotype dataset.

*Step 3: identify gender-mismatched subjects.* The gender of a subject can be inferred from genotypes of SNPs on chromosome X. In detail, for male samples, the homozygosity rate of SNPs on chromosome X is expected to be 1, since male only has one copy of X chromosome. However, the homozygosity rate is much lower for female samples. Comparing sex information in clinical records, the samples with discordant gender should be excluded, which might be due to sample contamination or plating errors [20]. In eQTLQC, the homozygosity rate of chromosome X is calculated based on all markers excluding the pseudo-autosomal region. And samples are labeled as female if homozygosity rate $< 0.2$, and labeled as male if homozygosity rate $>0.8$, empirically. Besides, eQTLQC requires at least 100 markers in default to enable this function. To be noted, the heterozygous haploid genotypes could be automatically set as missing in some genotype calling algorithms. In this case, eQTLQC cannot detect gender-mismatched samples based on homozygosity rate. In ROSMAP genotype data, all samples passed the gender checking.

*Step 4: set the heterozygous haploid genotypes as missing.* The variants on X chromosome in male samples should have haploid genotypes excluding the pseudo-autosomal region. The heterozygous haploid genotypes may exist in gender-mismatched samples, such as female sample labeled as male. After excluding gender-mismatched samples, described in *Step 3*, heterozygous haploid genotypes may be caused by sequencing or genotype calling errors, which should be removed. In this step, we set the heterozygous haploid genotypes as missing for all samples.

*Step 5: remove markers violating the principle of Hardy–Weinberg Equilibrium (HWE).* SNPs that have genotyping errors may significantly derive from the HWE and should be excluded. Given the frequencies of SNP alleles, allele A and B for instance, in a cohort, the expected frequencies of genotypes AA, AB and BB could be estimated under the HWE hypothesis. If the observed genotype frequencies of the SNP are far from the expected genotype frequencies, which could be evaluated by the Chi-squared test, we filter the SNP violating the principle of HWE. In eQTLQC, markers with HWE test *p* value $<10^{-6}$ will be identified and removed. In the ROSMAP dataset, 337,812 variants are removed in this step.

*Step 6: remove markers with informative missingness.* The failure of genotype calling may depend on genotypes, which can result in ’informative missingness’. For example, the successful calling rate of rare homozygous genotypes may be on average lower than heterozygous genotypes [19]. Bias will be introduced when estimating allele frequencies for SNPs with nonrandom missing genotypes [35]. In eQTLQC, we use the mishap test introduced in PLINK [34] to test whether the genotype missing status of a SNP can be predicted by neighbor SNPs. The mishap-test *p* value threshold is set to $10^{-9}$ by default in our pipeline, and 67 variants are removed in the ROSMAP genotype dataset.

*Step 7: remove markers with low minor allele frequency (MAF).* Due to factors like sample availability and cost, eQTL studies usually have sample sizes of dozens to thousands. In studies of limited sample size, variants with smaller MAF may not result in robust association signals. Typically, most eQTL studies apply MAF $\ge$ 1–10% depending on their sample size. And the MAF cutoff should be set higher for studies with smaller sample sizes. For example, MAF $\ge 0.1$ and 0.2 were applied in recent single-cell eQTL studies with sample size of 45 and 23 respectively [36, 37]. In eQTLQC, the default MAF lower bound is set to 5%. In ROSMAP dataset, 123,992 variants with MAF $\le 0.05$ are excluded in this step.

*Step 8: exclude subjects with outlying heterozygosity rate.* Heterozygosity rate is the proportion of heterozygous genotypes for an individual, which can reflect DNA sample qualities. Samples with excessive heterozygosity rates may be due to sample contamination, and samples with reduced heterozygosity rates may indicate sample inbreeding [19]. The distribution of mean heterozygosity rates in a cohort can be used to identify subjects with outlying heterozygosity rates. In eQTLQC, we use the mean ± 4 standard deviations of observed heterozygosity rates as the normal interval, and subjects with heterozygosity rates apart from the normal region will be excluded. Only independent SNPs are used for calculating heterozygosity rates. In the ROSMAP dataset, two outlying samples are removed in this step.

*Step 9: identify related and duplicated subjects. * Related and duplicated samples violate the prerequisites of eQTL mapping analysis where the linear regression model is usually used. Identification of related or duplicated subjects is based on two metrics: identity by state (IBS) and identity by descent (IBD). For each pair of subjects, the IBS score can be observed as 0, 1, and 2 at a given marker by counting the number of shared alleles. In other words, IBS0 represents two different alleles, and IBS1 and IBS2 represent 1 and 2 common allele(s) respectively. The shared allele(s) may be inherited from a recent common ancestor, and in this case, those shared alleles are called IBD. IBD can be estimated from genome-wide IBS scores [38]. The expectations of IBD equal to 1, 0.5, 0.25, and 0.125 for duplicates or monozygotic twins, first-degree relatives, second-degree relatives, and third-degree relatives, respectively. Since variation exists in practice due to population structures, genotyping errors, and complex LDs, the cutoff of IBD is slightly different. For individual pairs with IBD $>0.1875$ (second-degree relatives or closer), one subject is randomly removed; and for individual pairs with IBD $>0.98$ (duplicated samples or identical twins), both subjects will be excluded. In the ROSMAP dataset, six samples are excluded in this step.

**Fig. 3:**
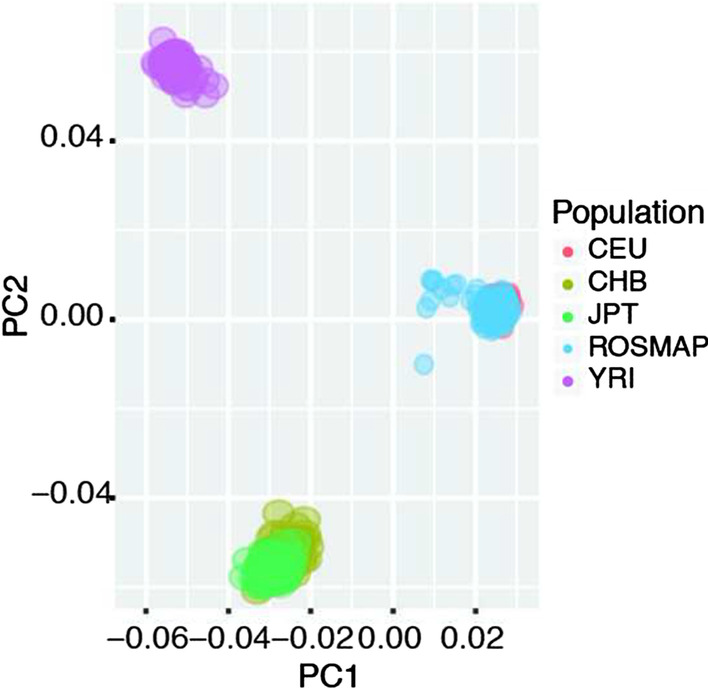
PCA. Population structure of ROSMAP cohort and HapMap cohorts

*Step 10: identify and exclude subjects with divergent ancestry.* Population stratification could bias the eQTL analysis as a major confounder, which needs to be adjusted by confounding adjustment approaches. Outlying subjects from divergent populations will enlarge the effect of population stratification and should be removed. To identify the population outlying samples, smartPCA [39] is employed in eQTLQC. To illustrate the population ancestry of ROSMAP samples, we first integrate ROSMAP genotypes and HapMap genotypes derived from four main populations: Yoruba trios from Ibadan, Nigeria (YRI), Utah residents of northern and western European ancestry (CEU), unrelated Japanese individuals from Tokyo, Japan (JPT) and Han Chinese individuals from Beijing, China (CHB). The top two principal components (PCs) are calculated from the integrated samples using LD-pruned SNP set and are shown in Fig. 3, where node color represents the population. As we can see, all samples in ROSMAP are classified into the CEU group. No population outliers were identified using SmartPCA. Besides, users can also select to output the top principal components derived from genotype profiles.

*Genotype imputation.* The genotype profiles that passed rigorous quality controls could be used for eQTL analysis. However, before preceding to eQTL analysis, genotype imputation is recommended for microarray-based genotype data, which usually has a limited number of markers. Excellent hidden Markov model (HMM)-based genotype imputation tools have been developed such as minimac3 [40], minimac2 [41], Beagle [42] and IMPUTE2 [43]. As the genotype imputation needs intense computational resources to store the genome reference panel and perform imputation jobs, we did not implement this function module into our automatic pipeline. Instead, we provided independent scripts for genotype imputation based on an online genotype imputation server, i.e., the Michigan Imputation Server (MIS) [40]. The MIS provides a user-friendly web interface, underlying which high-performance computing clusters are freely available. And several state-of-the-art human genome reference panels are hosted on the platform, such as HRC (consisting of 64,976 haplotypes) released by Haplotype Reference Consortium [44].

### eQTL mapping

334 subjects passed the quality controls in the preprocessing of both gene expression data and genotype data. 37.7% subjects are male, with an average age of 85.8, average RIN of 7.2 and average PMI of 7.5 h. 26,663 genes and 6,736,714 variants with MAF $\ge 0.05$ were used for mapping eQTL associations after the preprocessing (Table 1).

**Table 1:** Summary of eQTL mapping results. FDR is estimated using Benjamini–Hochberg procedure

Summary	Threshods	ROSMAP	Mayo	MSBB	CommonMind
Effective sample size	NA	334	103	65	200
Gene count	NA	26,663	27,101	29,736	21,511
Variant count	NA	6,736,714	6,932,711	6,937,313	6,703,202
Top PC count	NA	3	3	3	3
Cis-eQTL associations	$P<1$	124,877,625	131,696,349	143,560,218	103,667,853
Trans-eQTL associations	$P<10^{-4}$	18,617,087	19,594,408	21,324,856	14,353,808
Cis-eQTL associations	$FDR<0.05$	3,132,119	516,977	124,675	404,182
Cis-eQTL count	$FDR<0.05$	1,515,726	326,652	78,690	306,100
Cis-gene count	$FDR<0.05$	21,173	7999	3373	6021
Trans-eQTL associations	$FDR<0.05$	163,821	23,200	13,000	9151
Trans-eQTL count	$FDR<0.05$	78,894	13,607	6454	8914
Trans-gene count	$FDR<0.05$	3079	745	394	203

**Fig. 4:**
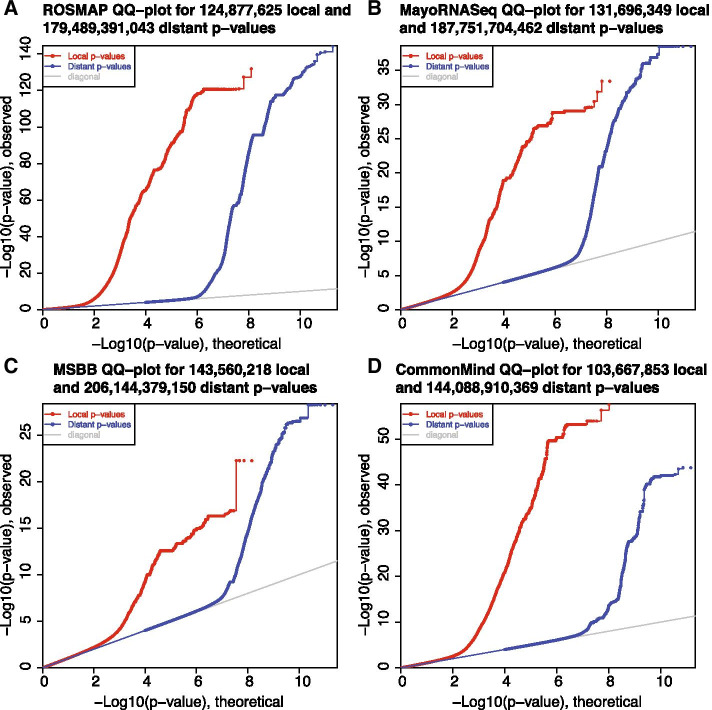
QQplot. Q–Q plots of local and distal eQTLs in ROSMAP, Mayo, MSBB and CommonMind studies. Theoretical (x-axis) *p*-values versus MatrixEQTL calculated *p*-values (y-axis) in $-\log 10$ transformation were plotted for each dataset. Red points represent cis-eQTLs, and blue points represent trans-eQTLs. The grey line represents null line

The final eQTL mapping analysis is conducted using R Package MatrixEQTL [16]. By default, the additive linear model will be applied. And in cis-eQTL analysis, SNPs are included if their positions are within 1Mb with the TSS of a gene. And trans-eQTL analysis includes SNP-gene associations if their distances are beyond this window. FDR reported by MatrixEQTL, using Benjamini–Hochberg procedure is used to measure the association significance. Besides, covariates could be adjusted in this step, such as the top PCs from genotype data, user-given confounders. In the ROSMAP dataset, we adjusted the top three PCs derived from genotype profiles since known and latent covariates related to gene expression profiles have been adjusted by SVA. 124,877,625 cis-associations and 179,489,391,043 trans-associations were tested, and the *p* value distributions were shown in Fig. 4. 1,515,726 cis-eQTLs and 78,894-trans-eQTLs were detected with FDR $\le 0.05$,associated with 21,173 local egenes and 3079 distant egenes, respectively.

### Robustness analysis

To evaluate the robustness of our pipeline, we conducted similar data preprocessing and eQTL mapping for the other three independent datasets: MayoRNAseq (Mayo) [24], MSBB [25] and CommonMind [26]. These datasets were also from brain tissues, and only neuropathologically healthy subjects were used for analyses, resulting in 103, 65 and 200 effective samples consisting of both gene expression and genotype data in each dataset respectively. The summaries of cis/trans-eQTL analyses are shown in Table 1. And the Q–Q plots of cis/trans-eQTL results of the four datasets are shown in Fig. 4.

**Fig. 5:**
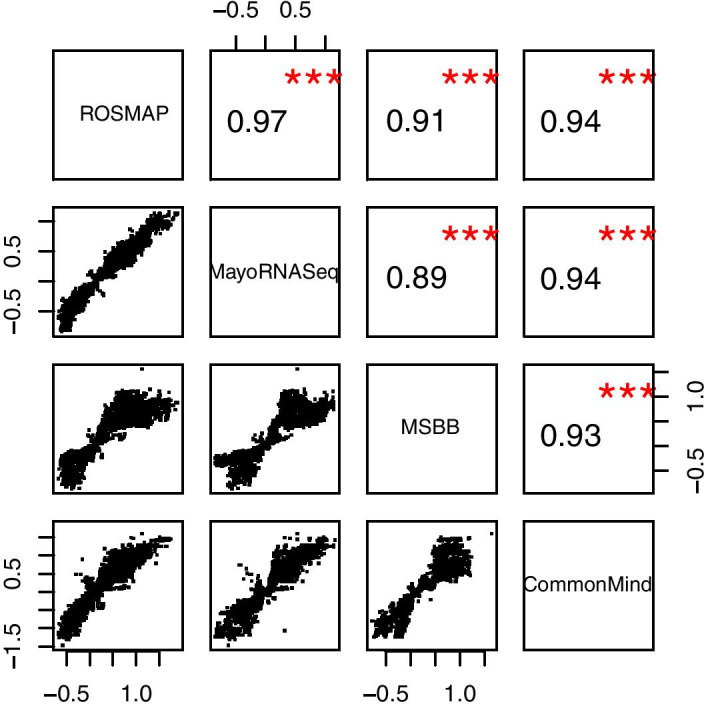
Betapairs. Effect sizes ($\beta$ values) of variant-gene pairs in eQTL results of ROSMAP, Mayo, MSBB and CommonMind studies

To further evaluate the concordance among independent datasets, we compared the effect sizes ($\beta$ values) of variant-gene pairs among these datasets. The lower side of Fig. 5 shows the $\beta$ values of all shared variant-gene pairs between datasets with *p* value less than 0.05. The upper side indicates the Pearson’s correlations of lower scatter plots, and the red star sign indicates the significance of correlation. We can see most Pearson’s r values are larger than 0.9, representing strong concordance. These results indicate that our pipeline could generate robust eQTL signals in different datasets.

## Conclusion and discussion

Advances in the development of tools and databases have accelerated the researches of complex diseases [45–47]. The eQTL analysis plays a key role in associating functional elements, such as coding or non-coding transcripts, with disease susceptibility variants. In this work, we propose eQTLQC, an eQTL analysis pipeline with automated preprocessing of both genotype data and gene expression data especially RNA-seq based. Our method aims to package the complex quality control, normalization, and eQTL mapping procedures required in eQTL analysis into a ’black box’ and leave users with a flexible interface to set up parameters and control the processing logic. For RNA-seq based gene expression data, eQTLQC accepts multiple data types and formats, such as Fastq, BAM, gene read count, and normalized metrics (e.g., TPM/FPKM/RPKM). Several main functional modules are followed, depending on the input data types, to transform the input data type into TPM metrics (or FPKM/RPKM if user provided). And rigorous quality control steps are applied to exclude problematic samples, such as gender mismatch, poor alignment. Data normalization and covariates adjustment are also applied to reduce data noises. For genotype data, eQTLQC support the widely-used VCF and PLINK formats. Ten rigorous quality control procedures are followed to exclude sample outliers and variant outliers. In our recent work [2], we have applied the core methods in eQTLQC to discover the genetic regulatory effects on both coding and non-coding transcripts. And in this work, we demonstrate its features and feasibility using the ROSMAP dataset. Furthermore, we demonstrated that eQTLQC is robust for eQTL mapping through case studies on multiple independent datasets.

Our work is also subject to several limitations. First, this pipeline has only been tested in the processing of the bulk RNA-seq dataset. For the single-cell RNA-seq based data, the data quality control and normalization methods are different from bulk RNA-seq, which should be further considered. Second, the order of some preprocessing steps is empirically set up. For example, the ’step 4: set the heterozygous haploid genotypes as missing’ should be downstream of ’step 3: identify and exclude subjects with mismatched sex’ as described in the Methods. However, the order of ’step 5: HWE-test’ and ’step 6: Mishap-test’ can be switched. The optimal preprocessing order is still an open question for discussion. Although the current study packages the complex processing details into a black box, users are limited to use the built-in methods. With the rapid revolution of bioinformatics methods and tools, how to maintain the tool up-to-date is also challenging.

In the future, we plan to integrate specific data processing techniques for single-cell RNA-seq based eQTL analysis and improve the user experience by designing Graphical User Interface(GUI).

## Methods

### Overview of eQTLQC framework

Two main phases are included in the eQTLQC framework: the data preprocessing phase and eQTL mapping phase, as shown in Fig. 1. The data preprocessing, which largely affects the accuracy and reliability of eQTL analysis, includes rigorous preprocessing steps for two main data categories needed for eQTL analysis, i.e., gene expression data and genotype data. As gene expression data has various data types and data formats, eQTLQC can handle the FASTQ format of raw reads, BAM format of mapped reads, read count data type of alignment summaries, and also normalized metrics such as RPKM, FPKM, and TPM. The read alignment module, feature-count module, and normalization module are executed depending on the imputed gene expression data formats (Fig. 1), resulting in a normalized gene expression matrix. Next, rigorous quality control steps will be applied to the normalized gene expression matrix to exclude outlying genes and samples. Then, quantile normalization and covariates adjustment (including known and hidden covariates) will be applied to the gene expression matrix to normalize gene expression profiles and adjust the gene expression biases caused by covariates, respectively.

In preprocessing of genotyping data, eQTLQC accepts widely used VCF and PLINK formats. Ten rigorous quality control steps are followed up to remove outlying variants and samples, including: (1) remove markers with excessive missing genotypes; (2) exclude subjects with excessive missing genotypes; (3) exclude subjects with gender-mismatch; (4) remove markers with heterozygous haploid genotypes; (5) remove markers violating Hardy-Weinberg Equilibrium (HWE); (6) remove markers with informative missingness; (7) remove markers with low minor allele frequency (MAF); (8) exclude subjects with abnormal heterozygosity rate; (9) exclude related subjects; and (10) identify and exclude individuals with divergent ancestry. After these rigorous quality control steps, clean genotyping data together with clean gene expression data will be reformatted to fit the requirement of MatrixEQTL, used in the eQTL mapping phase. In the following context, we will introduce the details of each step in the preprocessing of gene expression data and genotype data, and also the eQTL mapping phase based on real-world datasets generated by ROSMAP studies.

### Experimental datasets

The Religious Orders Study (ROS) [22] and Memory and Aging Project (MAP) [23] were two longitudinal cohort studies, aiming at studying aging and Alzheimer’s disease (AD). Participants enrolled in both studies were free of dementia at the beginning and agreed to longitudinal clinical recording and organ donation [48, 49]. CommonMind data were from CommonMind Consortium which provides large-scale, well-curated brain samples and relevant multi-omics datasets [26]. MayoRNAseq data were produced by Mayo Clinic Alzheimer’s Disease Genetics Studies (MCADGS) studying diseases such as AD, progressive supranuclear palsy (PSP) and pathologic aging (PA) [24]. MSBB study aimed at studying AD, and data were generated from postmortem brain tissue collected through the Mount Sinai VA Medical Center Brain Bank [25]. Post-mortem neuropathologic evaluations were all performed upon the death of participants. RNA-seq data, genotype data and clinical data derived from ROSMAP studies as well as MayoRNAseq, MSBB and CommonMind were downloaded from the Synapse platform (www.synapse.org).

## Data Availability

The datasets analyzed in the current study are available in the Synapse platform (https://adknowledgeportal.synapse.org/).

## Abbreviations

AD: Alzheimer’s disease
eQTL: Expression quantitative trait loci
FPKM: Fragments per kilobase million
GWAS: Genome-wide association studies
H-clustering: Hierarchical clustering
HMM: Hidden Markov model
HWE: Hardy–Weinberg equilibrium
IBD: Identical by descent
IBS: Identical by state
indel: Insertion and deletion
IQR: Interquartile range
MAF: Minor allele frequency
MAP: Memory and aging project
MIS: Michigan imputation server
NGS: Next-generation sequencing
PA: Pathologic aging
PMI: Post-mortem interval
PSP: Progressive supranuclear palsy
QC: Quality control
RIN: RNA integrity number
RLE: Relative log expression
ROS: Religious orders study
RPKM: Reads per kilobase million
SNP: Single nucleotide polymorphism
SVM: Support vector machine
TPM: Transcripts per kilobase million
WGS: Whole genome sequencing
